# Preliminary feasibility for recruiting and retaining black and white females to provide fecal samples for longitudinal research

**DOI:** 10.1186/s13099-019-0324-7

**Published:** 2019-08-26

**Authors:** Tiffany L. Carson, Rebecca B. Little, Sh’Nese Townsend

**Affiliations:** 1 Division of Preventive Medicine, Department of Medicine, School of Medicine, University of Alabama at Birmingham, 1720 2nd Avenue South MT 639, Birmingham, AL 35294-4410 USA; 2 Comprehensive Cancer Center, University of Alabama at Birmingham, Birmingham, AL USA; 3 Division of Preventive Medicine, Department of Medicine, School of Medicine, University of Alabama at Birmingham, 1720 2nd Avenue South MT 518K, Birmingham, AL 35294-4410 USA; 4 Division of Preventive Medicine, Department of Medicine, School of Medicine, University of Alabama at Birmingham, 1720 2nd Avenue South MT 518E, Birmingham, AL 35294-4410 USA

**Keywords:** Recruitment, Microbiome, Underrepresented minorities, Longitudinal, Biospecimen

## Abstract

As the associations between the gut microbiota and numerous health outcomes become more evident, it is important to conduct longitudinal microbiome research to advance the field beyond the identification of associations. It is also necessary to include individuals who have historically been underrepresented in biomedical research in longitudinal microbiome studies to better understand and eliminate racial/ethnic health disparities. This paper describes our experiences in recruiting and retaining participants for an ongoing, longitudinal microbiome study for which the main results will be reported at a later time. This article provides preliminary evidence of the feasibility of recruiting and retaining a racially diverse sample of females (97% completion for invited participants) for longitudinal microbiome research.

## Introduction

The gut microbiota is increasingly being identified as a contributor to health status. Greater microbial diversity has been associated with better health and the over- or underrepresentation of various taxa has been associated with a number of chronic conditions including obesity [1], some cancers [2], asthma [3], heart disease [4], and inflammatory bowel disease [5]. While the volume of studies identifying associations between the gut microbiota and a range of chronic conditions is great in number, cross-sectional studies alone do not allow for causal inference. Longitudinal studies are necessary to better elucidate whether gut microbial perturbations cause morbidity or occur as a result. For example, expert opinion in 2019 from the International Cancer Microbiome Consortium concluded that “data from longitudinal cohort studies are needed to confirm the role of the human microbiome as a key driver in the aetiopathogenesis of cancer [6]”.

In recent years, there has been an increased number of reports from longitudinal gut microbiome research with studies that ranged from samples collected over a 1-month period to 2 years. [7–28]. However, these studies generally did not report adequate representation of black females, a group that is historically underrepresented in biomedical research despite bearing a disproportionate burden of chronic diseases. Several of these studies focused on post-partum females, neonates and pediatrics [11, 13, 17, 22, 23, 25, 27, 28], transplant patients [15, 16, 19], or diseases including irritable bowel syndrome, *Clostridium difficile*, and HIV [8, 18, 20, 24, 26]. Only one study examining the gut microbiota among breast cancer survivors reported adequate representation of black females [9].

Longitudinal studies can present retention challenges for several reasons. Previous studies have cited participant-related factors, contextual and environmental factors, and research-related factors as contributors to attrition in research studies [29]. When considering participant-related factors (e.g., gender, income, influence of family members, emotional distress), black women may be at greater risk for refusal or attrition in a longitudinal follow-up study.

The purpose of this study was to assess the feasibility of recruiting a racially diverse sample of females to provide survey data, body measurements, and biospecimens (stool, blood, saliva) at baseline and at a 1-year follow-up. There was a particular focus on females and racial diversity in order to assess feasibility among groups historically underrepresented in biospecimen research. This paper describes our experiences in recruiting and retaining participants for an ongoing, longitudinal microbiome study for which the main results will be reported at a later time.

## Methods

As a part of a larger, ongoing cross-sectional study, a subgroup analysis of participants consecutively enrolled over a pre-defined 1 year period was conducted to assess the preliminary feasibility of establishing a longitudinal cohort of racially diverse females to participate in biospecimen research with a specific focus on the gut microbiome. In the main ongoing study, females who were non-Hispanic black (i.e., of African ancestry) or white (i.e., of European ancestry) were recruited to provide demographic, anthropometric, and survey data along with biospecimen (blood, stool, saliva) samples to examine racial differences in the gut microbiome and explore potential behavioral mediators (diet, stress). Individuals were excluded if they were younger than 19 years old, had taken antibiotics in the past 3 months, were pregnant or nursing at the time of screening, had a previous cancer diagnosis, reported a race other than black or white, or reported Hispanic ethnicity. Data were collected over 2 study visits approximately 1 week apart. At visit one, after completing informed consent, participants completed a demographics form, multiple psychometric surveys to assess stress, were measured for height and weight, and underwent venipuncture to provide a blood sample. Participants were also given a stool and saliva collection kit with written and verbal instructions about standardized collection, storage, and transportation methods for home self-collection. At visit 2, participants returned self-collected stool and saliva samples, completed a 24-h dietary recall using NCI ASA-24, a medications and supplements form, and completed a participant satisfaction survey. After completion of both study visits, participants were compensated $50. After all activities related to the main study were completed, participants were also asked to provide written consent if they were willing to be contacted for follow up research related to the main study. Research staff emphasized that the main study for which they had already consented to participate was completed and that participation in a follow up study was completely optional.

The subgroup analysis included all participants enrolled in the main study between October 2016 and August 2017. One to 2 weeks preceding the 1 year anniversary of completion of the main study, the study coordinator, who had already established a rapport and built trust with participants, attempted to contact participants who provided consent for follow-up to invite them back for an optional 1-year visit. Interested persons were screened by telephone to determine continuing eligibility for the follow up study. Interested and eligible participants were invited back to complete the same measures and provide the same bio-specimen samples that were collected during their initial participation in the main study. Our strategies to facilitate successful completion of the follow-up visits included leveraging established trust, full disclosure of study activities and the right to withdraw, and accommodation of participants’ schedule availability. Participants who completed both visits for the follow-up study were compensated an additional $50. Informed consent was obtained and all study activities were approved by the Institutional Review Board at the University of Alabama at Birmingham.

## Results

Of the 75 participants enrolled in the main study during the pre-specified time window, all but one provided consent to be contacted for follow up research. Contact was attempted for sixty-nine participants who are described in Table 1. On average, participants had a mean age and body mass index (BMI) of 45.2 years and 32.1 kg/m^2^, respectively. Black participants had significantly higher BMIs compared to whites (34.4 vs. 27.9 kg/m^2^; p < 0.01). Approximately half of participants reported a total annual household income of less than $39,999 and one-third were married (Table 1). The demographic and anthropometric trends of the subgroup mirror that of the study population of the larger main study (n = 190) at the time of this analysis (data not shown).

**Table 1:** Description of select characteristics of participants contacted for follow-up study (n = 69)

	Total (n = 69)	Black (n = 44)	White (n = 25)
	Mean ± SD	Mean ± SD	Mean ± SD
Age (years)	45.0 ± 13.1	43.7 ± 12.6	48.0 ± 14.0
Body mass index (kg/m^2^)	31.6 ± 7.8	33.9 ± 7.8	27.5 ± 5.8

	n (%)	n (%)	n (%)
Marital status^a^			
Single/never married	22 (32.4)	18 (41.9)	4 (16.0)
Married	23 (33.8)	10 (23.3)	13 (52.0)
Living with partner, but not married	2 (2.9)	1 (2.3)	1 (4.0)
No longer married (divorced, widowed)	20 (29.4)	13 (30.2)	7 (28.0)
Separated	1 (1.5)	1 (2.3)	0 (0)
Education			
High school/GED	8 (11.6)	7 (15.9)	1 (4.0)
Some college	18 (26.1)	14 (31.8)	4 (16.0)
Associate degree	10 (14.5)	8 (18.2)	2 (8.0)
Bachelor’s degree	20 (29.0)	9 (20.5)	11 (44.0)
Graduate or professional degree	13 (18.8)	6 (13.6)	7 (28.0)
Annual household income			
Less than $20,000	24 (34.8)	18 (40.9)	6 (24.0)
$20,000–$39,999	11 (15.9)	10 (22.7)	1 (4.0)
$40,000–$59,999	16 (23.2)	9 (20.5)	7 (28.0)
$60,000–$79,999	9 (13.0)	4 (9.1)	5 (20.0)
$80,000 or more	9 (13.0)	3 (6.8)	6 (24.0)

^a^Missing for n = 1

Of the 69 eligible participants that were attempted for the 1-year follow up study, we were able to reach 81%. After three telephone attempts and two written mailings, the remaining 19% were considered lost-to-follow-up. Of those who were reached, 97% agreed to return for a 1-year follow up visit. Reasons for declining included moving out of state (n = 1) and no longer interested in participating (n = 1). Although not powered to test for statistical differences, we conducted sensitivity analyses to better characterize participants who we were able to reach compared to those who we could not reach. Unreachable participants had lower BMIs (33.2 vs 27.0; p = 0.001) and tended to be older although the age difference was not statistically significant (46.5 vs 51.0 years; p = 0.13).

## Discussion

Our study findings provide preliminary support for the feasibility of establishing a racially diverse longitudinal cohort of females to participate in microbiome research that requires the collection of measurement and survey data as well as several biospecimen samples over multiple time points. The underrepresentation of minorities in biospecimen research is well documented and the call for increased enrollment has persisted for more than a decade. This work is consistent with and builds upon previous work from our team in which we demonstrated that black women are willing to provide biospecimen samples for research [30]. In our previous study, using community based participatory research methods, a sample of black females living in the Deep South provided saliva samples for research purposes. This study extends our previous work by demonstrating the ability to collect more invasive specimens like stool and blood in a clinical research setting.

Building trust is an important step to increasing diversity in biospecimen research and retaining participants. Along with displaying diversity among our team members, we also build trust with participants by openly communicating, fully disclosing each step in the research process, and describing exactly how samples will be used. Participants have regular contact with the study coordinator and are also reminded that they can withdraw from the study without penalty at any time and request that samples are destroyed. Our team also used visual models to demonstrate the collection process in order to build participant self-efficacy. Future research should be participant-centric with a particular focus on building trust among underrepresented populations. This study is limited by a small sample size, a limited follow-up time frame, and limited generalizability due to the inclusion of only females. However, the sample size and 1-year follow up period are adequate to provide preliminary feasibility and estimates about recruitment and retention yield to inform future research. While the exclusive focus on females prohibits inferences about males, our findings provide support for participation in longitudinal microbiome research among a population that has been historically underrepresented in biospecimen research.

It is a general consensus among experts that longitudinal studies are needed to vertically advance the field of microbiome research. It is additionally important that individuals who are typically underrepresented in research and often bear a disproportionate burden of morbidities are included in longitudinal microbiome studies. Our work suggests that a racially diverse sample of females can be successfully enrolled in biospecimen studies focused on the microbiome and are willing to participate in longitudinal follow up studies. These findings provide support for the design of longitudinal follow-up studies for microbiome research and the inclusion of diverse populations.

## Data Availability

The datasets used and/or analyzed during the current study are available from the corresponding author on reasonable request.
